# Progression of Aortic Arch Calcification Is Associated with Overall and Cardiovascular Mortality in Hemodialysis

**DOI:** 10.1155/2020/6293185

**Published:** 2020-06-20

**Authors:** Wei-Shiuan Chung, Ming-Chen Paul Shih, Pei-Yu Wu, Jiun-Chi Huang, Szu-Chia Chen, Yi-Wen Chiu, Jer-Ming Chang, Hung-Chun Chen

**Affiliations:** ^1^Department of Radiology, Kaohsiung Medical University Hospital, Kaohsiung Medical University, Kaohsiung, Taiwan; ^2^Department of Radiology, Kaohsiung Municipal Siaogang Hospital, Kaohsiung Medical University, Kaohsiung, Taiwan; ^3^Division of Nephrology, Department of Internal Medicine, Kaohsiung Medical University Hospital, Kaohsiung Medical University, Kaohsiung, Taiwan; ^4^Department of Internal Medicine, Kaohsiung Municipal Siaogang Hospital, Kaohsiung Medical University, Kaohsiung, Taiwan; ^5^Graduate Institute of Clinical Medicine, College of Medicine, Kaohsiung Medical University, Kaohsiung, Taiwan; ^6^Faculty of Medicine, College of Medicine, Kaohsiung Medical University, Kaohsiung, Taiwan; ^7^Faculty of Renal Care, College of Medicine, Kaohsiung Medical University, Kaohsiung, Taiwan

## Abstract

**Background:**

Vascular calcification is common and associated with unfavorable outcomes among patients with end-stage renal disease (ESRD). Nevertheless, little is known whether the progression of vascular calcification outweighs the baseline calcification in association with overall and cardiovascular (CV) mortality in hemodialysis (HD) patients.

**Methods:**

This study included 140 maintenance HD patients. Vascular calcification was assessed using the aortic arch calcification (AoAC) score measured from chest radiographs at the baseline and the second year of follow-up. Progression of vascular calcification (*Δ*AoAC) was defined as the difference between the two measurements of AoAC. The association of *Δ*AoAC with overall and CV mortality was evaluated using multivariate Cox regression analysis.

**Results:**

During the mean follow-up period of 5.8 years, there were 49 (35%) overall mortality and 27 (19.3%) CV mortality. High brachial-ankle pulse wave velocity was positively correlated with *Δ*AoAC, whereas old age was negatively correlated with *Δ*AoAC. In multivariate adjusted Cox analysis, increased *Δ*AoAC (per 1 unit), but not baseline AoAC, was significantly associated with overall mortality (HR, 1.183; 95% CI, 1.056–1.327; *p* = 0.004) and CV mortality (HR, 1.194; 95% CI, 1.019–1.398; *p* = 0.028).

**Conclusion:**

Progression of AoAC outperformed the baseline AoAC in association with increased risk of overall and CV mortality in HD patients. A regular follow-up of chest radiograph and AoAC score assessments are simple and cost-effective to identify the high-risk individuals of unfavorable outcomes in maintenance HD patients.

## 1. Introduction

Although advances in pharmaceutical and imaging technology improve precision of disease treatment and diagnosis, cardiovascular (CV) disease remains the major cause of morbidity and mortality among patients with end-stage renal disease (ESRD) [1–3]. The risk factors for CV disease in ESRD include traditional and nontraditional elements, such as diabetes, hypertension, dyslipidemia, fluid overload, endothelial dysfunction, inflammation, and vascular calcification [4–8]. Compared to the general population, patients with ESRD have a higher prevalence of vascular calcification [9, 10], and it has prognostic significance for CV outcomes in ESRD patients [11–14].

Vascular calcification is able to be detected using several imaging techniques, including computed tomography (CT), radiographs (such as plain radiographs of chest, lateral abdomen, pelvis, and hands), and ultrasonography [15–17]. Although cardiac CT can accurately access the vascular calcification, this diagnostic tool is costly and involves a certain degree of radiation exposure. The aortic arch calcification (AoAC) score is a simple, noninvasive, and semiquantitative assessment for evaluation of vascular calcification by chest radiograph [18]. It is also highly correlated with the AoAC volume as determined by multidetector CT [18]. Compared to the cardiac CT, consecutive measurements of AoAC from patients' radiographs are more practical in evaluation of the progression of vascular calcification. Among ESRD patients, several studies have shown the association between baseline vascular calcification and adverse outcomes [16, 19–22]. Furthermore, recent studies indicated the progression of vascular calcification as an independent predictor of mortality in patients undergoing HD or peritoneal dialysis [16, 23, 24].

Whether the progression of vascular calcification outweighs the baseline calcification in prediction of unfavorable outcomes remains uncertain in HD patients. Hence, this study is aimed at investigating the factors associated with the progression of AoAC and further exploring the baseline AoAC and progression of AoAC in association with overall and CV mortality in maintenance HD patients.

## 2. Materials and Methods

### 2.1. Study Patients and Design

**Figure 1:**
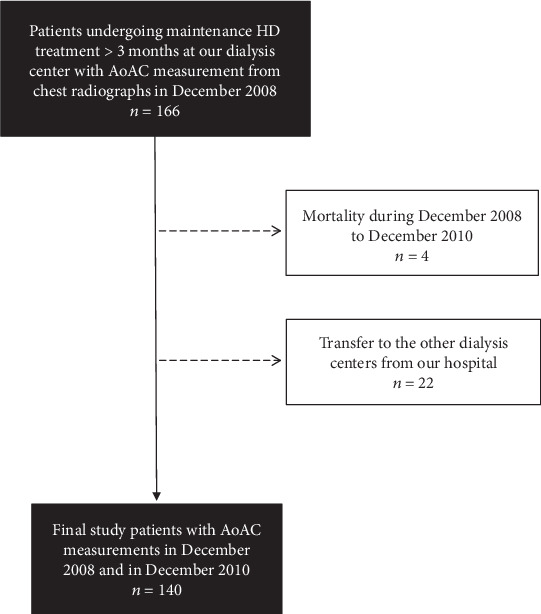
Flowchart of this study.

In this prospective study, we enrolled 166 patients undergoing thrice weekly maintenance HD treatment over 3 months at a dialysis center of a regional hospital in Taiwan at baseline in December 2008. The AoAC was measured from study patients' chest radiographs at the baseline and two years later. Of the 166 subjects, 140 (84.3%) completed the two AoAC measurements. Four patients died and 22 patients were transferred to other dialysis centers before the AoAC measurement in the second year. In total, 140 patients (59 males and 81 females; mean age 57.0 ± 13.6 years) were included (Figure 1). Compared to the included patients, the excluded patients had similar baseline characteristics, except for shorter hemodialysis vintage, higher prevalence of diabetes mellitus, higher level of fasting glucose, and lower level of serum creatinine. The study protocol was approved by the Institutional Review Board of Kaohsiung Medical University Hospital, and all participants provided their written informed consent. The methods were carried out in accordance with the approved guidelines.

### 2.2. Evaluation of AoAC and Cardiothoracic Ratio (CTR) by Chest Radiograph

One experienced and well-trained radiologist blinded to the study patients' clinical information reviewed their chest plain films. Calcification of the aortic arch was assessed using a scale, dividing the aortic arch on chest plain film into 16 sections by circumference [18]. The number of sectors with vascular calcification was counted in the 16 sections of the aortic arch to determine the AoAC score. CTR was defined using the chest radiographs, as the ratio of the transverse distance of the cardiac silhouette to the transverse distance of the chest.

### 2.3. Definitions of Change in AoAC (*Δ*AoAC) and Progression of AoAC

The *Δ*AoAC was defined as the AoAC score measured at the second year of follow-up minus the AoAC score measured at the baseline. ΔAoAC ≥ 1 was considered as a progression of AoAC.

### 2.4. Assessment of Ankle-Brachial Index (ABI) and Brachial-Ankle Pulse Wave Velocity (baPWV)

The ABI and baPWV were obtained 10 to 30 minutes before HD in patients' spine position using an ABI-form device, which measured blood pressure at arms and ankles simultaneously [25]. ABI was calculated as the ratio of the systolic blood pressure at the ankles divided by the systolic blood pressure at the arms. The baPWV value was automatically computed as the transmission distance divided by the transmission time.

### 2.5. Collection of Medical, Laboratory, and Demographic Information

We obtained the demographic and medical data as well as comorbid conditions from medical records and interviews with study patients. Blood samples were collected within one month of study enrollment. Laboratory data were measured from fasting blood samples using an autoanalyzer (Roche Diagnostics GmbH, D-68298 Mannheim COBAS Integra 400). The indicator of dialysis efficiency was evaluated using *Kt*/*V*, determined by the Daugirdas formula [26].

### 2.6. Definition of Overall and Cardiovascular Mortality

Overall and cardiovascular deaths were confirmed and ascertained from the medical records by two cardiologists, and disagreements were resolved through the adjudication of a third cardiologist. Patients were followed until death, and the remaining patients were followed until November 2018.

### 2.7. Statistical Analysis

Descriptive statistics were showed as percentages for categorical variables, means ± standard deviations for continuous variables with approximately normal distribution, or medians (25^th^–75^th^ percentile) for continuous variables with skewed distribution, such as *Δ*AoAC, HD vintage, serum triglycerides, intact parathyroid hormone (iPTH), and C-reactive protein (CRP). The differences between groups were checked by the Chi-squared test for categorical variables, by an independent *t*-test for continuous variables with approximately normal distribution, or by Mann-Whitney *U* test for continuous variables with skewed distribution. Significant variables (*p* < 0.05) in univariate analysis were selected for multivariate analysis. Multivariate adjusted linear regression analysis was used to identify the factors associated with the baseline AoAC and *Δ*AoAC. The continuous variables with a skewed distribution were log-transformed to attain normal distribution in the multivariate adjusted linear regression analysis. Furthermore, forward stepwise selection was used to include variables with entry probability < 0.05 to models for multivariate adjusted Cox proportional hazard analysis to identify the factors associated with overall and CV mortality. A *p* value < 0.05 was considered statistically significant. All statistical analyses were performed by SPSS software for Windows version 19.0 (SPSS Inc., Chicago, USA).

## 3. Results

A total of 140 patients with HD were enrolled with 42.1% men and 57.9% women. The mean age was 57.0 ± 13.6 years. Among study patients, 25.7% were with AoAC progression (*n* = 36).  Table 1 shows the comparison of characteristics among patients classified by AoAC nonprogression or progression. The median *Δ*AoAC for AoAC progression was 2. Compared to patients with AoAC nonprogression, those with AoAC progression tend to have higher baPWV, lower albumin, and lower creatinine levels.

### 3.1. Determinants of Baseline AoAC

Table 2 shows the determinants of baseline AoAC using multivariate adjusted linear regression analysis after adjusting for age; sex: smoking habit; history of diabetes, coronary artery disease, and cerebrovascular disease; pulse pressure; body mass index; ABI; baPWV; CTR; HD vintage; albumin; fasting glucose; serum triglycerides; total cholesterol; hemoglobin; creatinine; calcium-phosphate product; iPTH; CRP; *Kt*/*V*; calcium carbonate; and active vitamin D. Age (unstandardized coefficient *β*, 0.175; 95% confidence interval (CI), 0.112–0.238; *p* < 0.001), pulse pressure (unstandardized coefficient *β*, 0.049; 95% CI, 0.001–0.098; *p* = 0.046), HD vintage (unstandardized coefficient *β*, 2.222; 95% CI, 0.029–4.415; *p* = 0.047), calcium-phosphate product (unstandardized coefficient *β*, 0.096; 95% CI, 0.031–0.161; *p* = 0.004), and iPTH (unstandardized coefficient *β*, 1.785; 95% CI, 0.363–3.208; *p* = 0.014) were positively correlated with baseline AoAC.

### 3.2. Determinants of *Δ*AoAC

Table 3 shows the determinants of *Δ*AoAC using multivariate adjusted linear regression analysis after adjusting for demographic, clinical, and biochemical factors. Age (unstandardized coefficient *β*, -0.047; 95% CI, -0.092–-0.002; *p* = 0.043) was negatively and baPWV (unstandardized coefficient *β*, 0.123; 95% CI, 0.013–0.232; *p* = 0.028) was positively correlated with *Δ*AoAC.

### 3.3. Risk of Overall Mortality

The mean follow-up period was 5.8 ± 2.7 years for all patients. During the follow-up period, 49 deaths (35.0%) were recorded among these 140 patients, including CV deaths (*n* = 27), malignancy (*n* = 2), infectious disease (*n* = 11), gastrointestinal bleeding (*n* = 3), and others (*n* = 6).  Table 4 displays the hazard ratios (HR) of risk factors for overall mortality with adjustment for baseline AoAC, *Δ*AoAC, demographic, clinical, and biochemical factors and medication use. After multivariate forward analysis, increased *Δ*AoAC (per 1 unit; HR, 1.183; 95% CI, 1.056–1.327; *p* = 0.004), old age (per 1 year; HR, 1.091; 95% CI, 1.060–1.123; *p* < 0.001), cerebrovascular disease (HR, 2.705; 95% CI, 1.099–6.658; *p* = 0.030), decreased ABI (per 0.1 unit; HR, 0.826; 95% CI, 0.695–0.982; *p* = 0.030), and increased CRP (log per 1 mg/L; HR, 2.566; 95% CI, 1.393–4.727; *p* = 0.003) were associated with increased overall mortality.

### 3.4. Risk of CV Mortality

The documented CV deaths during follow-up included heart failure (*n* = 11), myocardial infarction (*n* = 4), ventricular fibrillation (*n* = 8), and hemorrhagic stroke (*n* = 4). A Cox proportional hazards regression analysis of risk factors for CV mortality is shown in Table 5. After multivariate forward analysis, increased *Δ*AoAC (per 1 unit; HR, 1.194; 95% CI, 1.019–1.398; *p* = 0.028), old age (per 1 year; HR, 1.061; 95% CI 1.023–1.010; *p* = 0.011), diabetes mellitus (HR, 2.809; 95% CI, 1.263–6.247; *p* = 0.011), and increased CRP (log per 1 mg/L; HR, 3.232; 95% CI, 1.437–7.267; *p* = 0.005) were associated with increased CV mortality.

## 4. Discussion

In the present study, the results highlight that increased *Δ*AoAC, but not the baseline AoAC, was significantly associated with overall and CV mortality during the mean follow-up period of 5.8 years among maintenance HD patients. Age, pulse pressure, HD vintage, calcium-phosphate product, and iPTH were positively correlated with the baseline AoAC. High baPWV was positively correlated with *Δ*AoAC, whereas age was negatively correlated with *Δ*AoAC.

The first important finding of the present study was that progression of AoAC at the 2-year follow-up outweighs the baseline AoAC on chest radiographs as a prognostic factor for overall and CV mortality in chronic HD patients. Jean et al. showed that the progression of vascular calcification at 3-year intervals is associated with mortality in 85 HD patients [27]. Sigrist et al. suggested that the progression of the calcification score of the femoral artery at 2-year intervals independently predicts death in 101 patients with stage 4 and 5 chronic kidney disease (CKD) [28]. Lee et al. demonstrated that the progression of AoAC at a 1-year interval is significantly associated with increased mortality among patients on peritoneal dialysis [16]. Taken together, our findings are in line with these studies reported above and indicate that simply assessing vascular calcification progression of a single site, the aortic arch, has prognostic implications.

Another important finding of our study showed baPWV has a significant positive correlation with *Δ*AoAC, but this correlation was not found with baseline AoAC after adjusting for potential confounders. The pathogenesis of vascular calcification progression is multifactorial and complex in ESRD [29]. Assessment of AoAC using chest radiographs cannot easily differentiate vascular calcification in two portions of the vessel wall, the intima and the media. We proposed that the baseline AoAC could not fully reflect the degree of medial calcification, which is the major driver of increased arterial stiffness in patients with chronic renal failure [30–32]. The transformation of vascular smooth muscle cells (VSMC) into osteoblast-like cells appears to be an essential element in the pathogenesis of medial calcification in the presence of calcium and phosphate deposition as well as uremic toxins [29, 33]. Our results suggested arterial stiffness as an independent risk factor for AoAC progression and could be possibly explained by patients with AoAC progression that might already have certain structural changes predominantly in the medial layer of the vessel wall. As a result, increased *Δ*AoAC is more ominous than the baseline AoAC in chronic HD patients.

Furthermore, we found that aging, HD vintage, pulse pressure, calcium-phosphorous product, and iPTH were positively correlated with the baseline AoAC. Although the present study is not aimed at investigating the whole scope of vascular calcification in ESRD, the abovementioned findings are consistent with previous studies [10, 18, 34]. Vascular calcification is now recognized as an actively cell-regulated pathology, including apoptosis and osteochondrogenic differentiation of VSMC, elastin degradation, and the release of extracellular vesicles loaded with calcium and phosphate [33, 35–37]. Certain active inhibitors protect vessels against abnormal mineral deposition under normal conditions. Among the dialysis-dependent population, dysregulation of calcium and phosphorous as well as iPTH acts as the active inducers of vascular calcification. Once the balance between active inhibitors and inducers of vascular calcification is broken, calcification can occur in the vessel walls. Raggi et al. demonstrated that cinacalcet plus low-dose vitamin D sterols could halt the progression of calcification in the coronary artery, aorta, and cardiac valve in secondary hyperparathyroidism patients undergoing HD [38]. Nevertheless, a prospective randomized trial showed that cinacalcet fails to reduce the risk of death or major CV events among HD patients [39].

The prognostic role of *Δ*AoAC superior to baseline AoAC and the independent correlation between baPWV and *Δ*AoAC were not well addressed previously. Although differences exist in the assessment of progression in vascular calcification, *Δ*AoAC in our study could reflect a more subtle change based on 16 grades of the AoAC score. There are some limitations to this study. First, the number of study patients is relatively small, and the outcome of 22 patients transferred to other facilities cannot be well evaluated. Second, certain confounding factors were not included in analyses, such as 25-hydroxy vitamin D, fetuin-A, and osteoprotegerin. Third, although the AoAC score is a simple, noninvasive, and semiquantitative tool for evaluating vascular calcification by chest radiograph and is highly correlated with AoAC volume by multidetector CT, it is less accurate in evaluating the extent of cardiovascular calcification than cardiac CT. Plain radiograph may not be sensitive enough to detect early-stage vascular calcification and subtle changes. The assessment may be affected by body size and overlapping structures. Fourth, we did not conduct imaging studies to assess the progression of abdominal aorta calcification. Finally, we did not have data of sevelamer and cinacalcet use, since they may have impacts on survival and slowing vascular calcification [40, 41].

## 5. Conclusion

Our study demonstrated that the progression of AoAC during a two-year follow-up in HD patients outperformed the baseline AoAC in association with increased risk of overall and CV mortality. A regular follow-up of the chest radiographs and AoAC score assessments are simple and pivotal to identify the high-risk individuals in this patient population.

## Figures and Tables

**Table 1:** Comparison of baseline characteristics in patients classified by AoAC nonprogression or AoAC progression.

Characteristics	All patients (*n* = 140)	AoAC progression (−) (*n* = 104)	AoAC progression (+) (*n* = 36)	*p*
Baseline AoAC	4 (1–8)	4 (0–8.75)	4 (2–6.75)	0.481
*Δ*AoAC	0 (-2, 1)	-1 (-2, 0)	2 (1, 3)	<0.001
Age (years)	57.0 ± 13.6	56.0 ± 14.0	59.8 ± 11.9	0.151
Men (%)	42.1	43.3	38.9	0.646
Hemodialysis vintage (years)	5.5 (3.2–8.4)	5.3 (3.1–8.4)	6.1 (3.4–8.5)	0.647
Smoking history (%)	24.3	25.0	22.2	0.738
Diabetes mellitus (%)	34.3	29.8	47.2	0.058
Coronary artery disease (%)	25.0	24.0	27.8	0.655
Cerebrovascular disease (%)	7.9	5.8	13.9	0.119
Pulse pressure (mmHg)	63.5 ± 16.2	61.9 ± 15.0	68.0 ± 18.5	0.051
Body mass index (kg/m^2^)	24.0 ± 3.5	24.0 ± 3.5	23.9 ± 3.6	0.829
ABI	1.10 ± 0.16	1.11 ± 0.16	1.10 ± 0.18	0.744
baPWV (cm/s)	1866.4 ± 515.8	1797.8 ± 472.0	2070.2 ± 589.6	0.017
CTR (%)	49.4 ± 5.5	49.1 ± 5.3	50.3 ± 6.1	0.292
Laboratory parameters				
Albumin (g/dL)	3.9 ± 0.3	3.9 ± 0.3	3.8 ± 0.2	0.046
Fasting glucose (mg/dL)	111.1 ± 40.8	108.1 ± 41.6	119.7 ± 37.5	0.147
Triglycerides (mg/dL)	125.5 (86–209.3)	127.5 (86–215.5)	122.5 (82.3–182.5)	0.453
Total cholesterol (mg/dL)	182.1 ± 39.7	185.5 ± 39.8	172.4 ± 38.2	0.088
Hemoglobin (g/dL)	10.1 ± 1.3	10.2 ± 1.3	10.0 ± 1.2	0.430
Creatinine (mg/dL)	10.8 ± 2.2	11.0 ± 2.3	10.1 ± 1.9	0.017
Calcium-phosphate product (mg^2^/dL^2^)	48.2 ± 12.2	48.6 ± 12.5	47.1 ± 11.3	0.528
Uric acid (mg/dL)	7.8 ± 1.6	8.0 ± 1.7	7.5 ± 1.5	0.136
iPTH (pg/mL)	438.6 (196.6–858.4)	447.5 (212.4–870.7)	353.1 (146.7–748.3)	0.426
CRP (mg/L)	0.26 (0.10–0.64)	0.27 (0.09–0.62)	0.26 (0.13–0.79)	0.502
*Kt*/*V* (Daugirdas)	1.6 ± 0.3	1.6 ± 0.3	1.6 ± 0.3	0.817
Medications				
Calcium carbonate (%)	74.3	76.0	69.4	0.441
Active vitamin D (%)	35.7	37.5	30.6	0.454

Abbreviations: AoAC: aortic arch calcification; ABI: ankle-brachial index; baPWV: brachial-ankle pulse wave velocity; CTR: cardiothoracic ratio; iPTH: intact parathyroid hormone; CRP: C-reactive protein. The *Δ*AoAC was defined as AoAC measured at the second year follow-up minus AoAC measured at the baseline. Patients with ΔAoAC ≥ 1 were considered as AoAC progression (+).

**Table 2:** Determinants of baseline AoAC using multivariate adjusted linear regression analysis.

Parameters	Multivariate adjusted	*p*
	Unstandardized coefficient *β* (95% CI)	
Age (per 1 year)	0.175 (0.112, 0.238)	<0.001
HD vintage (log per 1 year)	2.222 (0.029, 4.415)	0.047
Pulse pressure (per 1 mmHg)	0.049 (0.001, 0.098)	0.046
Calcium-phosphate product (per 1 mg^2^/dL^2^)	0.096 (0.031, 0.161)	0.004
iPTH (log per 1 pg/mL)	1.785 (0.363, 3.208)	0.014

Values expressed as unstandardized coefficient *β* and 95% confidence interval (CI). Adjusting for age; sex; smoking habit; history of diabetes, coronary artery disease, and cerebrovascular disease; pulse pressure; body mass index; ABI; baPWV; CTR; HD vintage; albumin; fasting glucose; serum triglycerides; total cholesterol; hemoglobin; creatinine; calcium-phosphate product; iPTH; CRP; *Kt*/*V*; calcium carbonate; and active vitamin D. Abbreviations are the same as Table 1.

**Table 3:** Determinants of *Δ*AoAC using multivariate adjusted linear regression analysis.

Parameters	Multivariate adjusted	*p*
	Unstandardized coefficient *β* (95% CI)	
Age (per 1 year)	-0.047 (-0.092, -0.002)	0.043
baPWV (per 100 cm/s)	0.123 (0.013, 0.232)	0.028

Values expressed as unstandardized coefficient *β* and 95% confidence interval (CI). Adjusting for age; sex; smoking habit; history of diabetes, coronary artery disease, and cerebrovascular disease; pulse pressure; body mass index; ABI; baPWV; CTR; HD vintage; albumin; fasting glucose; serum triglycerides; total cholesterol; hemoglobin; creatinine; calcium-phosphate product; iPTH; CRP; *Kt*/*V*; calcium carbonate; and active vitamin D. Abbreviations are the same as Table 1.

**Table 4:** Predictors for overall mortality using multivariate forward Cox proportional hazards model.

Parameters	Multivariate (forward)	*p*
	Hazard ratios (95% CI)	
*Δ*AoAC (per 1 unit)	1.183 (1.056–1.327)	0.004
Age (per 1 year)	1.091 (1.060–1.123)	<0.001
Cerebrovascular disease	2.705 (1.099–6.658)	0.030
ABI (per 0.1 unit)	0.826 (0.695–0.982)	0.030
CRP (log per 1 mg/L)	2.566 (1.393–4.727)	0.003

Values expressed as hazard ratios and 95% confidence interval (CI). Abbreviations are the same as in Table 1. Adjusting for baseline AoAC; *Δ*AoAC; age; sex; smoking habit; history of diabetes, coronary artery disease, and cerebrovascular disease; pulse pressure; body mass index; ABI; baPWV; CTR; HD vintage; albumin; fasting glucose; serum triglycerides; total cholesterol; hemoglobin; creatinine; calcium-phosphate product; iPTH; CRP; *Kt*/*V*; calcium carbonate; and active vitamin D. Forward stepwise selection included variables with entry probability < 0.05.

**Table 5:** Predictors for cardiovascular mortality using multivariate forward Cox proportional hazards model.

Parameters	Multivariate (forward)	*p*
	Hazard ratios (95% CI)	
*Δ*AoAC (per 1 unit)	1.194 (1.019–1.398)	0.028
Age (per 1 year)	1.061 (1.023–1.110)	0.001
Diabetes mellitus	2.809 (1.263–6.247)	0.011
CRP (log per 1 mg/L)	3.232 (1.437–7.267)	0.005

Values expressed as hazard ratios and 95% confidence interval (CI). Abbreviations are the same as in Table 1. Adjusting for baseline AoAC; *Δ*AoAC; age; sex; smoking habit; history of diabetes, coronary artery disease, and cerebrovascular disease; pulse pressure; body mass index; ABI; baPWV; CTR; HD vintage; albumin; fasting glucose; serum triglycerides; total cholesterol; hemoglobin; creatinine; calcium-phosphate product; iPTH; CRP; *Kt*/*V*; calcium carbonate; and active vitamin D. Forward stepwise selection included variables with entry probability < 0.05.

## Data Availability

The data supporting the findings of the present study are available within the article or are available from the corresponding authors upon reasonable request.
